# Evidence and implications of abnormal predictive coding in dementia

**DOI:** 10.1093/brain/awab254

**Published:** 2021-07-08

**Authors:** Ece Kocagoncu, Anastasia Klimovich-Gray, Laura E Hughes, James B Rowe

**Affiliations:** 1 Cambridge Centre for Frontotemporal Dementia, Department of Clinical Neurosciences, University of Cambridge, Cambridge CB2 0SZ, UK; 2 Medical Research Council Cognition and Brain Sciences Unit, University of Cambridge, Cambridge CB2 7EF, UK; 3 Cambridge University Hospital NHS Foundation Trust, University of Cambridge, Cambridge, CB2 2QQ, UK; 4 Basque Centre on Cognition, Brain and Language, San Sebastian 20009, Spain

**Keywords:** predictive coding, dementia, top-down processing, prediction, neurodegeneration

## Abstract

The diversity of cognitive deficits and neuropathological processes associated with dementias has encouraged divergence in pathophysiological explanations of disease. Here, we review an alternative framework that emphasizes convergent critical features of cognitive pathophysiology. Rather than the loss of ‘memory centres’ or ‘language centres’, or singular neurotransmitter systems, cognitive deficits are interpreted in terms of aberrant predictive coding in hierarchical neural networks. This builds on advances in normative accounts of brain function, specifically the Bayesian integration of beliefs and sensory evidence in which hierarchical predictions and prediction errors underlie memory, perception, speech and behaviour. We describe how analogous impairments in predictive coding in parallel neurocognitive systems can generate diverse clinical phenomena, including the characteristics of dementias. The review presents evidence from behavioural and neurophysiological studies of perception, language, memory and decision-making. The reformulation of cognitive deficits in terms of predictive coding has several advantages. It brings diverse clinical phenomena into a common framework; it aligns cognitive and movement disorders; and it makes specific predictions on cognitive physiology that support translational and experimental medicine studies. The insights into complex human cognitive disorders from the predictive coding framework may therefore also inform future therapeutic strategies.

## Teaser

Kocagoncu *et al*. propose an alternative framework for interpreting the cognitive deficits observed in dementia. They argue that deficits should be considered in terms of aberrant predictive coding in hierarchical neural networks, rather than as reflecting the loss of functional hubs or singular neurotransmitter systems.

## Introduction

Cognitive deficits in neurodegenerative diseases have often been characterized as the loss of core functional modules in distinct brain regions, or specific networks, each serving functionally specialized cognitive systems such as memory, language comprehension or executive function. This approach emphasizes the functional differences between disorders linked to functional anatomical susceptibility and network vulnerability.^1^ Alongside these functional anatomical differences that contribute to distinct phenotypes, preclinical models and clinical studies suggest convergence in important aspects of the pathophysiology of different dementias, with commonalities for example in terms of loss of synapses, synaptic plasticity and major neurotransmitters.^2^ The relative contributions of toxic misfolded protein aggregates, neuroinflammation and proteostasis to synaptic impairment vary across dementias, but their physiological consequences overlap, with potential convergence on a core cognitive mechanisms of predictive coding. Here we propose a re-evaluation of the diversity of cognitive features in dementia, in terms of impairments in predictive coding, leading to a trans-diagnostic neuro-computational model that may aid the development of novel therapeutic strategies.

Predictive coding is a core feature of brain function, implementing generative models that ‘explain’ sensory inputs via hierarchical beliefs about the world.^3-6^ In this review, we reassess clinical deficits in terms of the disruption of predictive coding in precisely tuned neural hierarchies engaged in prediction, prediction error and inference. The predictive coding account of normative brain function integrates cognitive and computational neuroscience to explain perception and action. The central tenet is that the brain acts as an active inference machine that learns statistical regularities of the external world (Box 1) and generates predictions to increase the efficiency of information processing and understanding of the sensorium.^3–6^

**Box 1 Predictive coding and hierarchical networks**

Predictive coding is a process by which the brain updates a model of the environment, to explain sensory inputs. The process applies hierarchically over increasingly abstract causes, and over time, forming the basis of diverse cognitive and behavioural functions. It rests the premise that perception is a probabilistic inference. Complex and abstract beliefs are represented in higher levels (e.g. on semantics and social norms) and direct sensory inputs at lower levels. Based on learned statistical dependencies, each level predicts the activity in the level below (‘feedback’). A mismatch between the prediction and the sensory input leads to a prediction error, which is propagated back up the hierarchy (‘feedforward’). The forward and backward connections convey prediction errors and predictions, respectively.^7^^,^^8^

Different biological implementations of predictive coding have been put forward at micro- and macroscopic levels,^3–6^ but they have multi-level hierarchies of neural circuits in common. There are different algorithmic implementations of the way in which the fit between predictions and sensory data is optimized, and the underlying model updated (e.g. linear estimation of parameters,^6^ Bayesian inference,^9^ a review of models).^10^ There are also alternatives to predictive coding, that nonetheless posit that the brain performs a probabilistic inference in hierarchical networks, and maintains a generative (i.e. explanatory) model of the environment by alternative mechanisms.^11^ This review does not seek to differentiate these alternative mechanisms, but focus on their commonalities, with the generation of predictions and updating them in response to prediction errors.

A critical feature of predictive coding is the estimation of uncertainty of the predictions and sensory inputs. Both the predictions and prediction errors are relayed with varying ‘precision’ (i.e. the inverse of variance, or uncertainty). This precision determines the relative weighting of the prediction error, whilst priors are updated iteratively, across all levels of the hierarchy.^12^^,^^13^ Precision weighting of the prediction errors is controlled by neuromodulation (Box 2) and postsynaptic gain control at the cellular level. Feedforward propagation of more precise prediction errors will have a greater impact updating beliefs represented in the higher levels (i.e. faster learning). Feedback generation of more precise predictions ‘cancels out’ incoming prediction errors, leading to stable beliefs and behaviour (i.e. slow learning). Healthy cognition requires fine tuning of this process, adjusting relative precision at upper versus lower levels of the hierarchy. The impact of neurodegeneration on the neural mechanisms that regulate precision, and govern the representations within each level, explain diverse cognitive and behavioural phenomena in dementia, and raise new hypotheses about candidate treatment strategies.

**Box 2 Precision changes in dementia and neurotransmitters**

‘Precision’ represents the level of certainty, and describes the confidence attributed to prediction errors at each level of the cortical hierarchy.^3^^,^^5^ For example, in noisy settings with high levels of uncertainty (e.g. driving on a foggy day, talking during a concert), precision of the sensory prediction errors is reduced while the precision at the higher levels is relatively increased. Neurotransmitters such as acetylcholine,^14–18^ glutamate,^19–21^ GABA^22–27^ and norepinephrine^18^ have been shown to regulate prediction errors and their precision across different cortical hierarchies. Impairments in the neurotransmitter mediated precision weighting gives rise to diverse clinical representations in dementia depending on the level and the functional domain of the cortical hierarchy where the mechanistic impairment occurs.

**Figure 1:**
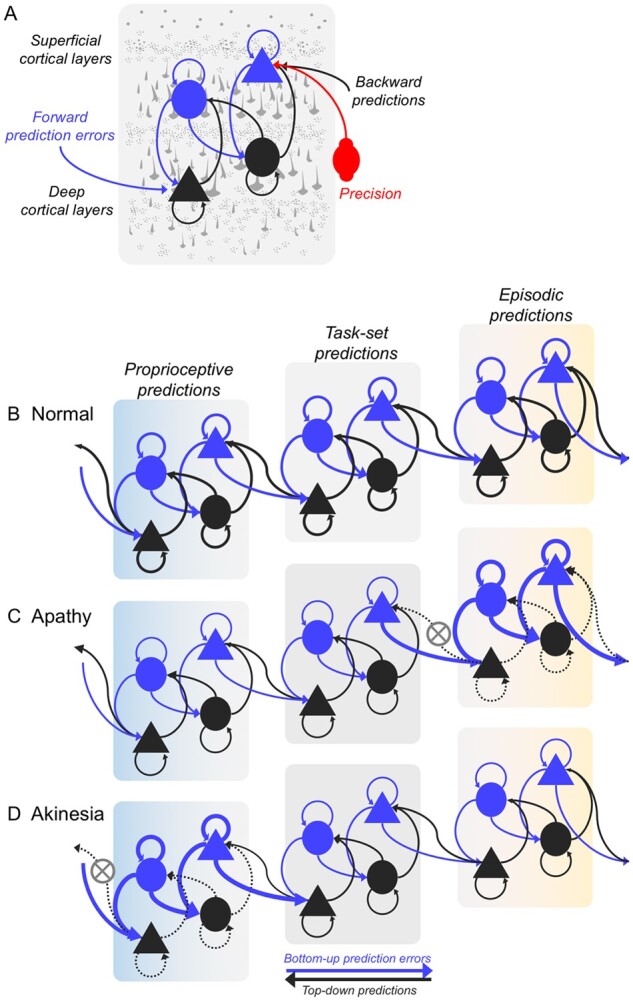
**Predictive coding mechanism within the hierarchical brain network.** (**A**) Schematic illustration of the predictive coding mechanism in a single cortical region at one layer in the hierarchy. Top-down predictions are conveyed via the backward connections (black arrows) from state representation units (black nodes) in deep cortical layers. The predictions are compared with conditional expectations at the lower level in the hierarchy by the error units in the superficial cortical layers (blue nodes) to produce prediction errors, which are passed bottom-up (blue arrows) to the higher level to update the predictions. Triangles and circles represent pyramidal neurons and inhibitory interneurons respectively. Precision weighting (red) regulates the postsynaptic gain of the error units, e.g. via neuromodulation. Panels **B**–**D** illustrate three layers of a hierarchical network of the behavioural/motor system, with three cortical layers from *left* (light blue) to *right* (yellow). Each layer of the hierarchy makes predictions relayed in a top-down fashion. Higher layers of the network make episodic predictions that are multimodal, abstract and span across a longer timescale (e.g. ‘that the city marathon is happening’). Intermediate layers represent medium-term, task-set or context specific predictions (e.g. ‘I am running, and see supporters and water stands’). Lower layers make transient, proprioceptive predictions on the immediate consequences of running action (e.g. ‘position of my limbs’). (**B**) Healthy state of the hierarchy with optimal control in which top-down predictions are matched by sensory inputs, minimizing prediction errors at each layer. In apathy and akinesia, behavioural impairments arise from a mismatch between the strength of predictions and prediction errors. (**C**) In apathy, top-down predictions at the higher level are represented with insufficient precision, and are therefore overwhelmed by bottom-up prediction errors from the intermediate hierarchical level. Therefore, high-level priors, representing abstract goals and desires, fail to be translated into specific proprioceptive predictions for movement, and as such there is a loss of goal-directed behaviour. (**D**) In contrast, with akinesia there is a poverty of movement because predictions at the lowest hierarchical level fail to suppress proprioceptive prediction errors. Even though the absence of behaviour may manifest similarly in apathy and akinesia, the underlying mechanism of impairment arises from predictive mismatch in different levels of the hierarchical network.

An example of the abnormally high precision in the lower levels of the hierarchy comes from Parkinsonian disorders. Akinesia, the poverty of movement, can arise from reduced precision in the higher order sensorimotor prediction errors, and an over-reliance on sensory evidence (Fig. 1D).^12^^,^^28^ Akinesia can be partially improved using the peripheral vibration devices that increase the uncertainty of sensory evidence, thereby reducing the precision.^29^^,^^30^ However precision changes are more commonly observed at the higher levels of the hierarchy. In normal ageing, impairments in vision and hearing, lead to the adaptation of precision weights across the cortical hierarchy,^31^ where the reliance on ‘inaccurate’ sensory evidence is reduced, and to balance, precision at higher cortical levels are boosted. Similarly, in Parkinson’s disease and Lewy body dementia, in the visual cortical hierarchy, the precision at the higher level prediction errors are up-weighted, albeit abnormally, giving rise to visual hallucinations.^32–36^

A key modulator of precision is acetylcholine that suppresses prediction errors at the higher order and regulates precision of the sensory prediction errors.^14–17^ Cholinergic loss can affect ascending sensory precision even in the absence of atrophy. Impaired mismatch negativity responses in Alzheimer’s disease, indicating unsuccessful sensory learning, is partially explained by the widespread degeneration of cholinergic projections.^37^^,^^38^ Similarly, patients with Lewy body dementia who have more severe degeneration of their cholinergic pathways experience more visual hallucinations.^39–41^ Cholinesterase inhibitors that mediate sensory precision, can amplify the amplitude of the mismatch response in patients with Alzheimer’s disease,^42^ and alleviate hallucinations in Lewy body dementia.^43^ Acetylcholine regulates inhibitory activity by suppressing or inactivating GABAergic interneurons.^25–27^ While slower neurotransmitters like acetylcholine are proposed to compute the precision, faster neurotransmitters like GABA are thought to encode the prediction errors.^44^ Patients with behavioural variant frontotemporal dementia show reduced mismatch negativity response, as a product of impaired inhibitory connections and reduced GABA concentrations in the frontal cortex.^23^^,^^24^ These patients show reduced precision in higher levels of the auditory hierarchy, leading to errors in encoding of conditional expectations at lower levels.^22^

The predictive coding account provides a common neurobiological framework to describe diverse cognitive, perceptual and behavioural phenomena. For example, there is evidence for predictive coding in vision,^45^^,^^46^ rhythm perception,^47^^,^^48^ auditory processing,^49–53^ reward and preferences^54^ and action control.^55^^,^^56^ The representation of predictions, prediction errors and precision in each system depends on a fine-tuned cortical hierarchy, with laminar-specific connectivity and balanced excitatory-inhibitory neurochemistry (Fig. 1A). Deficits in predictive coding have been proposed to cause domain-specific and domain-general cognitive impairments in neuropsychiatric disorders as diverse as psychosis,^57^^,^^58^ autism^59^^,^^60^ and alien limb.^61^

We propose that dementias’ effects on memory, perception, language and action control may also arise from a change in predictive coding. In particular, we set out how the effect of neurodegeneration on the ‘precision’ of predictions and prediction error can impair perception, learning and complex behaviours. The symptoms arising from a change in predictive coding are a function of the neural networks that are selectively vulnerable to each specific molecular pathology. The predictive coding account of dementia is therefore not an alternative to network specificity models,^1^ but instead augments these models by describing the homologous changes in predictive coding arising within each network.

We start with the basic processes of perception and action to introduce the principles predictive coding and the direct evidence is strongest. We then consider higher cognitive disorders, of amnesia and aphasia, and neuropharmacological factors, with examples drawn from studies of Alzheimer’s disease, Parkinson’s disease, frontotemporal dementia and dementia with Lewy bodies.

## Perception

In perceiving our environment, one makes use of prior knowledge and context to predict sensory inputs. For example, in a complex auditory scene such as a noisy cocktail party, prior knowledge or experience facilitates the parsing of constituent objects (or speakers) in time and space, making it easy to recognize one’s own name (‘the cocktail party effect’).^62^ Top-down predictions based on prior experience of the speakers, their language and the topic, facilitate this segregation.^63^ In vision, context-based predictions likewise aid rapid object recognition under both normal and challenging conditions.^4^^,^^64^ The use of auditory predictions is largely preserved in normal ageing. Indeed, people may become more dependent on their predictions and perceptually less sensitive to the sensorium with age, as the precision of the higher-order prediction errors increases relative to the precision sensory evidence.^14^^,^^28^

This balance is disrupted in mild cognitive impairment and dementia, with degeneration of temporo-parietal cortex from Alzheimer’s disease.^65^ Accordingly, patients develop greater difficulty following conversations in the presence of background noise, show impairments in segregating, tracking and grouping auditory objects that evolve over time^66^ and in perceiving sound location and motion.^65^ They become worse even at automatic prediction of repetitive stimuli and fail to generate a prediction error following unexpected sensory events. This failure to generate a prediction error with Alzheimer’s disease and other dementias is readily seen in the reduced ‘mismatch negativity responses’ in oddball tasks.^37^^,^^67–69^ Alzheimer’s disease similarly impairs higher order precepts such as melodic contours.^70^ Even otherwise healthy *APOE4* carriers (i.e. at an elevated risk of developing Alzheimer’s disease) show impairments in detecting auditory targets using contextual information.^71^

In the visual domain, hallucinations and illusions commonly occur with cortical Lewy body pathology, in Parkinson’s disease dementia and dementia with Lewy bodies. The perceptual content is commonly influenced by the immediate environment or autobiographical memories, with pareidolic experiences in ambiguous scenes,^72^ or the perception of familiar people or pets even if known to have died.^73^ The hallucinations are typically visually complex and familiar.^14^^,^^15^^,^^74^ This can be understood as a result of abnormal up-weighting of beliefs (i.e. more precise priors) that establish overly precise predictions relative to down-weighting (i.e. less precise) visual sensory evidence.^32–34^^,^^58^ Note that it is not just the absolute precision that matters, but the relative precision between upper and lower levels in a hierarchy. Note too that the symptoms depend on the anatomical distribution of the network that represents the cognitive hierarchy. The medial temporal and medial prefrontal areas are implicated in the cognitive hierarchy for such misperceptions,^75^ with hallucinations associated with abnormal activity and connectivity among lower visual cortical regions.^35^^,^^76–83^ The loss of cholinergic modulation of the precision of neural representations is a candidate cause, even in the absence of significant atrophy. Such cholinergic loss reduces the precision of feed-forward prediction errors relative to the precision of feedback predictions from higher level priors.^14–17^ This accords with the observation that patients who have more severe degeneration of their cholinergic pathways experience more visual hallucinations,^39–41^ and symptoms are alleviated with cholinesterase inhibitors.^43^

## Action, apathy and behavioural disorder

As Adams *et al*.^84^ highlight, perceptual and motor systems are not separate entities, but operate as a single ‘inference machine’ that serves to predict sensory input in all sensory domains and intermediate inferences on the causes of the sensory inputs. The concept of ‘active inference’ posits that prediction errors can be reduced by actively changing sensory inputs through movement. Active inference uses hierarchical predictive coding, with direct evidence coming from the physiology of motor control (Fig. 1A).^56^

The failure to attenuate proprioceptive prediction errors in the lower levels of a behavioural hierarchy leads to akinesia (Fig. 1D),^85^ in the context of neurodegenerative movement disorders like Parkinson’s disease. Over-precise priors (in upper levels of a motor control hierarchy, represented by premotor and prefrontal cortex) also explain the alien limb syndrome (that one’s own limb is moving without intention or volition). Specifically, alien limb syndrome is associated with disrupted information flow between medial areas (supplementary motor area) that encode precision of proprioceptive predictions to the lateral pre-motor areas which encode action outcomes.^61^

There is empirical evidence for active inference at the lower level of the cognitive hierarchy for behaviour, expressed as specific actions. For example, there is ubiquitous ‘sensorimotor attenuation’ in health across the lifespan: a transient down-weighting of the predicted sensory consequences of actions, observed in 98% of healthy adults (Fig. 1B).^31^ Attenuation facilitates movement and provides a sense of agency.^86^ In healthy ageing, there is greater reliance on predictions arising from greater precision of prior beliefs, and less on the sensorium.^31^ In neurodegenerative parkinsonism, deficits in sensorimotor predictions (reduced precision) results in an over-reliance on sensory evidence and poverty of movement.^12^^,^^28^ Such deficits in sensorimotor predictions are linked to disease severity of corticobasal syndromes,^28^^,^^86^ and to atrophy and white matter connectivity of the pre-supplementary motor area—a cortical region that lies at the intermediate level of a spatially embedded cognitive hierarchy for behaviour, between motor cortex and prefrontal cortex.^28^^,^^40^

There are therapeutic implications of active inference. For example, akinesia can be improved by high frequency peripheral vibration which reduces the precision of sensory evidence and increasing the relative precision of sensorimotor predictions (*cf.* Sweeney *et al*.^29^ and Macerollo *et al*.^30^). This is in line with suggestions that high-frequency vibration attenuates proprioceptive feedback allowing for greater top-down control.^87^ A physiological correlate is the decrease of power of beta oscillations at the onset of the vibration, preceding the improved movement. Similar beta desynchronization^13^^,^^88^^,^^89^ is essential for movement planning and initiation.^90^ In bradykinetic disorders, beta power is elevated,^91–94^ while dopaminergic treatment in Parkinson’s disease enhances beta desynchronization,^93^^,^^95^ alleviates akinesia, and increases sensorimotor attenuation.^28^^,^^96^ Under active inference, beta power may index somatosensory precision and therefore mediate sensorimotor attenuation, modulated by dopamine.^28^^,^^96^

A lack of behaviour can also be caused by apathy, without akinesia. Apathy is common in dementia, including Alzheimer’s disease, dementia with Lewy bodies, frontotemporal dementia and vascular dementia.^97–99^ We propose that apathy arises from deficits in the precision of the higher order predictions of goal-states and context rather than proprioception (Fig. 1C). This is analogous to the causes of akinesia, but at a higher level of a cognitive hierarchy for goal-directed behaviour.^100^ When the relative precision of the goal prior is low, it will fail to propagate through the hierarchy down to effector mechanisms, and the outcome is a lack of behaviour.^57^^,^^85^^,^^101^ The failure of active inference thereby shifts from lack of movement (akinesia) to a lack of goal-directed behaviour (apathy) according to the level of the hierarchy in which precision is affected by the cellular and pharmacological effects of each molecular pathology.

In healthy controls, trait apathy is associated with lower precision of predictions about action outcomes.^100^ In dementia-related apathy, there is limited direct evidence for higher variance of priors, but indirect support comes from the failure to modulate prefrontal cortical beta oscillations in goal-directed tasks and the correlation between challenging everyday behaviours and beta-power (specifically, the failure of task-related beta-desynchronization).^102^ We suggest an anatomical correlate of goal priors lies in anterior cingulate and medial prefrontal cortex, with loss of connectivity to motor cortex and the striatum.^86^^,^^103–105^

Disinhibited and impulsive behaviours are common to many dementias,^98^^,^^106^^,^^107^ with a predisposition to act out of context, prematurely, or on the basis of little evidence.^108^ Such behaviours would be explained by impaired precision of high-order predictions which diminish the confidence weighting on the choices or behavioural policies available. This can lead to ‘jumping to conclusions’.^109^ Dopamine dysregulation may explain some types of impulsivity (e.g. Parkinson’s disease^110^), but other neurotransmitters such as noradrenaline, GABA, and glutamate, modulate behavioural control and are also deficient in many neurodegenerative disorders.^2^ For example, noradrenaline regulates impulsive behaviour via widespread projections from the locus coeruleus to the cortex,^111–113^ in response to salient cues that trigger shifts in behaviour.^114^ In the predictive coding framework, the locus coeruleus noradrenergic signals update predictions at higher levels mediated by fronto-striatal circuits, in response to prediction error (e.g. ‘surprise’).^115^ The locus coeruleus is affected by Alzheimer’s disease, Parkinson’s disease and frontotemporal lobar degeneration,^111^ which has led to noradrenergic treatment strategies to reduce impulsivity.^116–118^ In active inference terms, behaviours become impulsive and inflexible when the precision of priors is not updated in response to salient behavioural cues.

## Memory and learning

Memory deficits and poor learning are prominent features of dementia, including but not limited to Alzheimer’s disease. The degeneration of the medial temporal lobe may affect memory retrieval and associative learning in part because of the disruption of predictive coding in these circuits. The hippocampus encodes expectancies of future events based on the probabilistic consequences of past events,^119–121^ and hippocampal activity is modulated by the predictability of the future events.^122^ Hippocampus not only encodes individual episodes but also the ordinal structure of events, a distributed in space, time (time in relation to internal computational demands, not an external clock) or other properties. The representation of ordinal structure may appear as encoding sequences or locations, but it can also be seen as part of a more fundamental generative model of the environment—an ‘inference machine’ engaged in predictive coding.^123^^,^^124^ Such a hippocampal-based hierarchy operates over multiple timescales.

**Figure 2:**
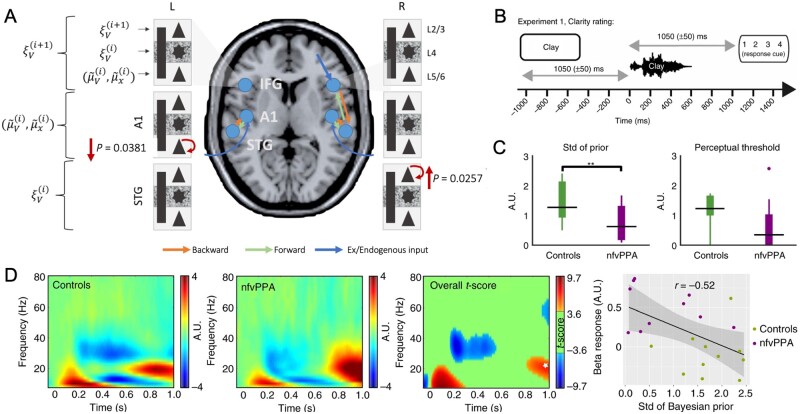
**Neurophysiological changes associated with predictive coding impairments.** (**A**) Cortical microcircuit dynamic causal model of the mismatch negativity response in patients with behavioural variant frontotemporal dementia, compared with healthy controls. Local (intrinsic) decreases in self-modulation of the deep pyramidal cells in the primary auditory cortex (A1), and increases in self-modulation of the superficial pyramidal cells in the superior temporal gyrus, lead to failure to establish sensory predictions and thereby reduced mismatch response. (**B**) Illustration of the MEG paradigm used by Cope *et al*.,^186^ in which participants were presented with a written word followed by a noise vocoded spoken word that either matched or mismatched with the written word. Participants rated the clarity of the spoken words. (**C**) Derived parameters from Bayesian data modelling show that patients with non-fluent primary progressive aphasia (nvPPA) had more precise priors (smaller variance) than controls. A.U. = arbitrary units. (**D**) Induced responses between the cue offset and spoken word onset: beta power was higher in the nvPPA group after 800 ms and negatively correlated with precision of the prior expectations. **A** is reprinted from Shaw *et al*.^22^ with permission. **B**–**D** are reprinted from Cope *et al*.^186^ with permission.

The ability to anticipate events over very short timescales is impaired by many dementias. For example, oddball tasks such as the auditory mismatch negativity paradigm have been interpreted to rely on short term ‘memory traces’ for sensory events. Such tasks have provided some of the strongest direct evidence for predictive coding.^14^^,^^125–129^ The mismatch response indexes the prediction error, that is fed-forward in a frontotemporal hierarchy to update predictions that are in turn fed backwards.^126^ The active nature of auditory predictions has been corroborated by computational and dynamic causal modelling. Simulations show that the mismatch response is an output of active cortical predictions rather than passive synaptic habituation.^128^ Omitted events in mismatch paradigms provide an ideal test of cortical hierarchies that actively predict events. Indeed, dynamic causal modelling of omitted events show increased connectivity from and to the prefrontal cortex similar to the connectivity changes observed for the mismatch stimuli.^130^ In dementia, the mismatch negativity amplitude is reduced,^69^^,^^131^^,^^132^ together with impaired frontotemporal connectivity (Fig. 2A).^22^^,^^69^^,^^133^^,^^134^ Patients with Alzheimer’s disease show larger reductions at longer inter-stimulus intervals^37^^,^^67^^,^^135^ in relation to reduced temporal activity and cognitive score of executive function.^131^^,^^136^

Patients with Alzheimer’s disease have difficulty encoding and processing novel information (e.g. high rates of false recognition of novel items,^137^^,^^138^ reduced primacy,^139^^,^^140^ von Restorff effect^141^) associated with reduced functional connectivity between hippocampus, temporal and frontal areas.^142^ Asymptomatic *APOE4* carriers compared to non-carriers, show reduced prediction errors to novel words, and elevated hippocampal activity to subsequently remembered words.^143^ In those at risk of familial Alzheimer’s disease, *PSEN1* and *APP* mutation carriers who approach the familial age of diagnosis, show elevated blood oxygenation level-dependent response in the middle temporal gyri during novelty encoding.^144^ These impairments in novelty processing are consistent with impaired predictive processing in a hippocampal hierarchy. Larger prediction errors generated after encountering novel or contextually unexpected items (e.g. ‘the butcher in the *office*’), drive stronger episodic encoding compared to expected items (e.g. ‘the butcher in the butcher *shop*’).^145^^,^^146^ Unsuccessful learning could therefore result from smaller prediction errors arising from relatively low precision weighting of the prediction error.^145^^,^^147^

At the cellular level, the modulation of the precision of a hippocampal prediction error in memory tasks is dependent on both cholinergic and dopaminergic modulation of NMDA receptor plasticity^14^^,^^148–151^ Impaired mismatch response in Alzheimer’s disease is partially explained by the degeneration of cholinergic projections, in the presence of relatively preserved top-down propagation of predictions from intact higher level priors.^136^ Cholinergic agents partially restore the mismatch response in Alzheimer’s disease,^42^ enhancing feed-forward signalling by precision of the sensory evidence weighting.^14^^,^^152^ Similarly, dopamine is proposed to modulate saliency of the stimuli in hippocampus in response to novelty and facilitate encoding of the new information via its connections with the ventral tegmental area and substantia nigra.^150^^,^^151^^,^^153–155^ Supporting this, administration of dopamine agonists, accelerates the processing speed of novel information,^156^ and enhances recollection.^157^ GABAergic modulation of feedback predictions and feedforward prediction errors may also contribute to the impairment of predictive coding from frontotemporal lobar degeneration.^23^^,^^24^

## Speech and language

In health, language comprehension shows remarkable speed and resistance to noisy environments. This is enabled by predictive coding at multiple levels of linguistic representation: phonological,^158–160^ semantic,^161–166^ syntactic^167–169^ and discourse context.^170^ In neurodegenerative aphasias, poor comprehension arises from the impact of lesions on the frontotemporal and temporo-parietal networks which support top-down propagation and updating of predictions. For example, people with non-fluent variant primary progressive aphasia show particular vulnerability to processing deficits and delays at the lexical level when speech inputs are degraded^171^^,^^172^ or ambiguous.^173–175^ This arises from degeneration of frontal and perisylvian cortex, with reduction of top-down control used to optimize perception and production of speech,^176–179^ leading to speech production deficits and agrammatism,^180–182^ In contrast, damage to the temporo-parietal junction leads to speech repetition deficits^183^^,^^184^ arising from disrupted mapping between priors for speech representations and proprioceptive articulatory predictions in the ventral motor cortex and inferior frontal cortex.^84^^,^^185^

Cope *et al*.^186^ showed that in the presence of intact temporal cortex, frontal lobe neurodegeneration from non-fluent variant primary progressive aphasia causes overly precise contextual priors, together with reduced frontal-to-temporal directional connectivity in the beta frequency range (Fig. 2B–D). This combination leads to delayed resolution of speech inputs by the temporal cortex, and impaired perception of degraded speech input. The reliance on overly precise priors explains the paradoxical relative advantage for patients as noise increases (in contrast to healthy adults). The patients’ speech comprehension deficit was more severe in quiet settings. Overly precise priors may also affect speech production in primary progressive aphasia: whereas delayed auditory feedback in healthy controls reduces fluency and accuracy of speech,^187^^,^^188^ delayed feedback does not impair fluency. This suggests a reliance on internal models of speech and relative weakness of the precision of sensory representations.^189^

Efficient reading requires top-down signalling from higher order language areas, to disambiguate visually confusable words.^190^ While damage to the left medial occipito-temporal areas causes alexia and object agnosia with spared central language abilities and orthographic knowledge,^191^^,^^192^ reading deficits are often more severe than object recognition deficits. Lesions of inferior frontal cortex cause auditory agnosias and pure word deafness.^193^^,^^194^ Woodhead *et al*.^195^ showed that whole-word training to improve reading was associated with stronger feedback connectivity from the inferior frontal gyrus to the occipital areas, and bidirectional connectivity between ventral occipito-temporal and occipital areas. This suggests stronger top-down priors aid prediction of the words in reading.

Semantic processing of words in context is similarly dependent on top-down signalling, as contextual information and prior knowledge is used to predict forthcoming words.^165^^,^^196^^,^^197^ The N400 is an electrophysiological index of the prediction error, reflecting the degree of mismatch between semantic priors and sensory input.^198^ In semantic dementia differentiation of concepts that belong to the same semantic category is impaired, such as ‘giraffe’ and ‘zebra’ (i.e. taxonomic blurring). The N400 is absent for mismatches of the same semantic category,^199^ suggesting that semantic priors are under-specified (i.e. imprecise). Furthermore, disambiguating meaningful objects (but not meaningless shapes) in difficult viewing conditions is also impaired,^200^ suggesting a domain-general deficit of top-down semantic control, thought to depend on intact connectivity within the larger fronto-temporo-parietal network.^201^

## Conclusion

We propose a reformulation of cognitive deficits in dementia away from specific localized functional-anatomical impairments towards a generalized framework of aberrant Bayesian inference, within cortical hierarchies. Predictive coding principles can be generalized to account for multiple cognitive and perceptual impairments observed in neurodegenerative diseases, arising from diverse molecular aetiologies. The cognitive deficits and related neurophysiological abnormalities, can be understood in terms of altered precision in the normally finely-balanced feedforward and feedback pathways in cortical hierarchies. There are multiple potential cellular and molecular pathological routes to disrupt the precision of predictions and prediction errors, including localized cell loss (atrophy), and changes in acetylcholine, dopamine, and noradrenaline, that weight the importance (i.e. precision) of predictions and gain function of prediction errors. The predictive coding framework provides a unifying framework to understand the effects of these changes, in different hierarchical functional brain networks, as the basis for different dementia syndromes. It is a powerful trans-diagnostic framework that can aid better understanding of the mechanisms of disease across the lifespan and in turn facilitate new therapeutic strategies for dementia. New analytical methods enable new experimental medicine studies with techniques like dynamic causal modelling that can inform the efficacy and mechanism of candidate therapies. We therefore hope that this Update on predictive coding stimulates progress towards a new form of precision medicine, defined in terms of the precise cognitive and physiological consequences of disease.

## Funding

E.K. is funded by the Dementias Platform UK and Alzheimer’s Research UK (RG94383/RG89702). J.B.R. is supported by the Wellcome Trust (103838) and Medical Research Council (SUAG/051 G101400) and the National Institute for Health Research Cambridge Biomedical Research Centre. L.E.H. is funded by the Wellcome Trust (103838). A.K.-G. is funded by the European Union’s Horizon 2020 Research and Innovation Programme under the Marie Skłodowska-Curie grant (798971).

## Competing interests

J.B.R. received research funding from Janssen, Lilly, AstraZeneca and GSK in the past 3 years, and is acting as a consultant for SV Health, Astex, Biogen, UCB, Curasen, Asceneuron, and Alzheimer Research UK, none related to the current work.
